# Update on Intravenous Immunoglobulin in Neurology: Modulating Neuro-autoimmunity, Evolving Factors on Efficacy and Dosing and Challenges on Stopping Chronic IVIg Therapy

**DOI:** 10.1007/s13311-021-01108-4

**Published:** 2021-11-11

**Authors:** Marinos C. Dalakas

**Affiliations:** 1 Department of Neurology, Thomas Jefferson University, Philadelphia, PA USA; 2 Neuroimmunology Unit, Dept. of Pathophysiology, National and Kapodistrian University of Athens Medical School, Athens, Greece

**Keywords:** Intravenous immunoglobulin, Neurological disorders, Immunomodulation by IVIg, Initiating and stopping IVIg, IVIg and IgG4 neuro-autoimmunities

## Abstract

In the last 25 years, intravenous immunoglobulin (IVIg) has had a major impact in the successful treatment of previously untreatable or poorly controlled autoimmune neurological disorders. Derived from thousands of healthy donors, IVIg contains IgG1 isotypes of idiotypic antibodies that have the potential to bind pathogenic autoantibodies or cross-react with various antigenic peptides, including proteins conserved among the “common cold”-pre-pandemic coronaviruses; as a result, after IVIg infusions, some of the patients’ sera may transiently become positive for various neuronal antibodies, even for anti-SARS-CoV-2, necessitating caution in separating antibodies derived from the infused IVIg or acquired humoral immunity. IVIg exerts multiple effects on the immunoregulatory network by variably affecting autoantibodies, complement activation, FcRn saturation, FcγRIIb receptors, cytokines, and inflammatory mediators. Based on randomized controlled trials, IVIg is approved for the treatment of GBS, CIDP, MMN and dermatomyositis; has been effective in, myasthenia gravis exacerbations, and stiff-person syndrome; and exhibits convincing efficacy in autoimmune epilepsy, neuromyelitis, and autoimmune encephalitis. Recent evidence suggests that polymorphisms in the genes encoding *FcRn* and *FcγRIIB* may influence the catabolism of infused IgG or its anti-inflammatory effects, impacting on individualized dosing or efficacy. For chronic maintenance therapy, IVIg and subcutaneous IgG are effective in controlled studies only in CIDP and MMN preventing relapses and axonal loss up to 48 weeks; in practice, however, IVIg is continuously used for years in all the aforementioned neurological conditions, like is a “forever necessary therapy” for maintaining stability, generating challenges on when and how to stop it. Because about 35-40% of patients on chronic therapy do not exhibit objective neurological signs of worsening after stopping IVIg but express subjective symptoms of fatigue, pains, spasms, or a feeling of generalized weakness, a conditioning effect combined with fear that discontinuing chronic therapy may destabilize a multi-year stability status is likely. The dilemmas of continuing chronic therapy, the importance of adjusting dosing and scheduling or periodically stopping IVIg to objectively assess necessity, and concerns in accurately interpreting IVIg-dependency are discussed. Finally, the merit of subcutaneous IgG, the ineffectiveness of IVIg in IgG4-neurological autoimmunities, and genetic factors affecting IVIg dosing and efficacy are addressed.

## Introduction

The last 25 years, intravenous immunoglobulin (IVIg), a pooled of polyclonal IgG from thousands of donors, has had a breakthrough impact in the treatment of autoimmune neurological disorders. Since first approved by regulatory agencies for Chronic Inflammatory Demyelinating Polyneuropathy (CIDP) in 2008, approval was subsequently granted for treating Guillain–Barre syndrome (GBS) and multifocal motor neuropathy (MMN) and since July 15 2021 for dermatomyositis [1–3]. Apart from approved indications however, IVIg has been effective in various immunologically diverse acute and chronic neurological disorders, including myasthenia gravis, inflammatory myopathies, stiff-person syndrome, neuromyelitis spectrum disorders, or autoimmune encephalitis, based on controlled clinical trials or large-scale studies. Such an overwhelming success, along with its easy access and excellent safety profile, has however led to its liberal use generating concerns as to whether IVIg is overused even for putatively neuro-autoimmune or poorly understood conditions. Most importantly, we have been witnessing its continuous use for chronic therapy, without evidence-based data, as several patients become conditioned being very reluctant to stop it due to fear that their chronic stability status might be disturbed.

The need for an updated review of IVIg is now appropriate not only in addressing the aforementioned practical issues but also in highlighting progress in understanding its mechanistic effects on specific immune pathways, the factors that affect the half-life of infused IgG or its reduced efficacy, and the need for dose adjustments as “one dose may not fit all.” Accordingly, this timely review is aimed to summarize the advances in understanding the evolved key immune factors targeted by IVIg and the various natural neuro-autoantibodies within the IVIg preparations, some of which are of practical relevance today in the SARS-CoV-2 pandemic; highlight the benefit, or lack thereof, based on new controlled trials; identify the reasons for not being effective in some patients within the approved indications, like IgG4 antibody–mediated conditions or genetic variables affecting the catabolism of infused IgG; point out biologic factors that influence dosing or efficacy; and address practical issues on IVIg administration including switching to subcutaneous routes. Most importantly, the review addresses the emerging issues on how and when to stop chronic IVIg therapy to avoid overuse highlighting that in approximately 40% of patients after long-term use, there is a significant conditioning effect requiring periodic assessments to ensure its judicious use.

## What is IVIg and What Does It Contain?

### Contents and Safety of Transmitting Pathogens

Each batch of IVIg is made by cold ethanol fractionation (Cohn’s process) of human plasma derived from pools of 5000–10,000 healthy donors [4–6] after having been deprived of coagulation factors. The product is purified by enzymatic treatment at low pH, followed by fractionation and chromatography [6, 7]. Treatment with solvent detergents at low pH, virus filtration, or the addition of caprylate, a naturally occurring fatty acid widely distributed in animal and vegetable fats, are additional powerful measures of filtering out viruses reducing even the probability of transmitting prions [7]. Within the final product, the purified immunoglobulins are stabilized with either glucose, maltose, glycine, mannitol, albumin, or l-proline that also ensures long shelf-life of the products at room temperature and concentrations up to 20% IgG [7, 8]. The marketed IVIg contains more than 95% IgG, less than 2.5% IgA, and negligible amount of IgM [4, 8]. Among the IgG subclasses, IgG1 varies from 55 to 70%, IgG2 from 0 to 6%, and IgG4 from 0.7 to 2.6%, according to the size and composition of the donor pools used in various proprietary IVIg preparations [4–9]. Not all the IVIg preparations are the same in reference to osmolality, stabilizing agents, content of IgA, subclasses of IgG, pH, and formulation (lyophilized or liquid forms) [9]. There is no however evidence that the biological action is different among the various commercial products, although their tolerance or allergic reactions may differ among recipients.

### Idiotypic and Neutralizing Antibodies Within the IVIg Preparations

The IgG of normal humans contains low-titer antibodies against a wide spectrum of proteins and anti-idiotypic antibodies, directed against Fαβ, the antigen-binding region of these autoantibodies [4–6, 9, 10]. Because IVIg preparations are derived from a large pool of human donors, the IgG molecules within the IVIg contain antibodies with a wide range of idiotypic and anti-idiotypic specificities [5, 6, 10–12]. While the anti-idiotypic IgG antibodies in an individual donor are circulated in a monomeric form, the IgG antibody complexes within the IVIg form dimeric pairs [6]. On electron microscopy, 40% of the IgG molecules form dimers connected by double-arm or single-arm binding between their F(αβ′)_2_ domains [10]. The larger the pool of donors, the higher the number of F(αβ′)_2_ dimers and the wider their spectrum of idiotypic-anti-idiotypic specificities [10–12]. Keeping the dimer content below a threshold level through dissociation ensures reduction of adverse reactions.

IVIg also contains neutralizing antibodies against epitopes of superantigens as well as antibodies against the Vβ_3_, Vβ_8_, and Vβ_17_ gene families of the T-cell receptor peptides [13]. Superantigens (i.e., bacterial toxins, enterotoxins, viruses) stimulate a large fraction of Vβ chain-expressing unsensitized T-cells and increase cytokine secretion that may play a role in breaking tolerance or triggering relapses in certain neurological diseases like MG, CIDP, or GBS [14]. IVIg neutralizes superantigens, as shown in Kawasaki’s disease [15], and could prevent the activation or clonal expansion of superantigen-triggered cytotoxic T-cells. Such an effect may be relevant in explaining the effect of IVIg in inhibiting relapses triggered by infections as seen in MG, relapsing GBS/CIDP or NMO-SD.

### Natural Neuronal Autoantibodies Within the IVIg Preparations: Significance in IVIg-Infused Patients

Sampling of various commercially available IVIg preparations with cell-based assays (CBA), ELISA, and immunohistochemistry on sciatic nerve and brain sections revealed the presence of several neuronal antibodies [16]. Among those tested, anti-GAD antibodies were detected with ELISA—but not CBA—in low (< 2000 IU/ml) titers, as typically seen in Type-I diabetes, but not > 2000 IU/ml, as seen in GAD-spectrum neurological disorders [17]. Anti-AQP4 antibodies were also detected by ELISA in most IVIg preparations in titers seen in AQP4-seropositive NMO-SD; these antibodies were not however detected with CBA and immunohistochemistry, indicating that they are directed against linear, rather than structural, epitopes, as part of the natural immune repertoire [16, 18]. Some IVIg samples also contain antibodies against MAG and HMGCR, but not against MOG and NMDAR [16]. This information is clinically important when testing sera from patients who have received IVIg to avoid false positivity with disease-specific autoantibodies. Consistent with the half-life of IgGs, these antibodies are not detected in the patients’ serum after 4–6 weeks from IVIg therapy.

### Anti-SARS-CoV-2 Antibodies Within IVIg: Cross-Reactivities with Pre-pandemic Seasonal Coronaviruses

Some IVIg preparations from pre-pandemic healthy donors exhibit cross reactivities with SARS-CoV-2 S1 antigens, with some preparations having clinically significant antibody titers as high as those seen in COVID-19-infected patients [19]. These observations highlight that the respective healthy IVIg donors had either natural autoantibodies or cross-reactive antibodies against antigenic protein fragments conserved among the other “common cold”–related coronaviruses, similar to the cross-reactivity observed for T-cells and B cells from COVID19-unexposed donors [20]. Whether these antibodies have any protective or therapeutic effects in COVID-19-exposed neurological patients with autoimmune diseases, currently receiving monthly IVIg maintenance therapy, is unclear. IVIg has been however effective in COVID-19-immune inflammatory syndrome resembling Kawasaki’s disease [21], while a randomized placebo-controlled trial has demonstrated that in severe COVID-19-infected patients, the in-hospital mortality rate was significantly lower in the IVIg-treated group compared to controls [22]. Apart from any potential benefit that remains still unproven until ongoing randomized trials are completed, the information that IVIg preparations contain such antibodies is important when assessing SARS-CoV-2 seroprevalence or vaccination-triggered antibodies to dissect whether SARS-CoV-2-antibodies detected in the sera of IVIg-treated patients are derived from IVIg or from acquired antiviral humoral immunity [19].

### Kinetics of Infused IgG and CSF penetrance: Relevance to En-bathing roots, Nerve End-plates, and Muscle Tissues

**Fig. 1:**
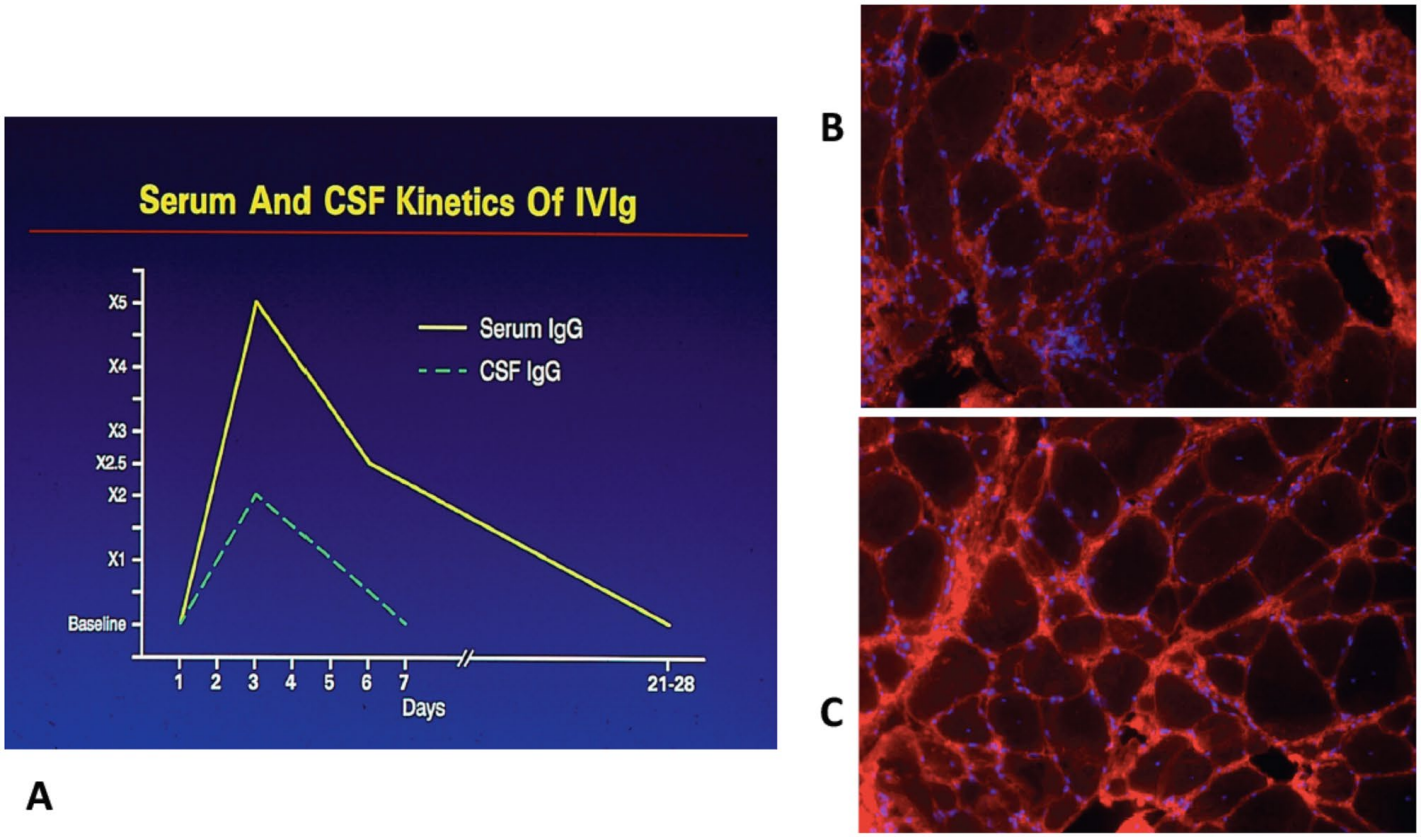
**A**–**C** Kinetics of infused IVIg: penetrance into CSF and muscle tissue. **A** Serum and CSF IgG, 3 days after infusion of IVIg. IgG increases fivefold in serum and twofold in the CSF. Rapid diffusion into the extravascular space follows [25]. **B**, **C** Muscle biopsies from a patient with IBM immunostained with human anti-IgG antibodies before IVIg (**B**) and after IVIg administration (**C**). Abundance of the infused IgG is detected in the endomysium, including around muscle fibres and inter-fascicular septae [170]

After an intravenous infusion of 2 g/kg IVIg (the standard initiating therapeutic dose), the serum IgG levels increase fivefold but decline by 50% in 72 h returning to pretreatment levels after 21–28 days similar to half-life of the native IgG [6, 23–25] (Fig. 1A). The marked initial decrease reflects extravascular redistribution. During the first 48 h of the infusion when serum IgG level is high, IgG also enters the cerebrospinal fluid where its concentration increases as much as twofold, returning to normal within a week [24, 25] (Fig. 1A). The infused IVIg also enters freely within the muscle, detected around muscle fibers and endomysial tissue (Fig. 1B, C), en-bathing the end-plate region, but also the nerves and roots that lack blood-CSF barrier; whether these direct tissue contacts exert any local anti-inflammatory effects on muscle and nerve tissues, relevant to inflammatory myopathies and neuropathies, complementing the systemic immunomodulatory actions, is however unknown.

### Mechanisms of Actions of IVIg in Autoimmune Neurology

**Fig. 2:**
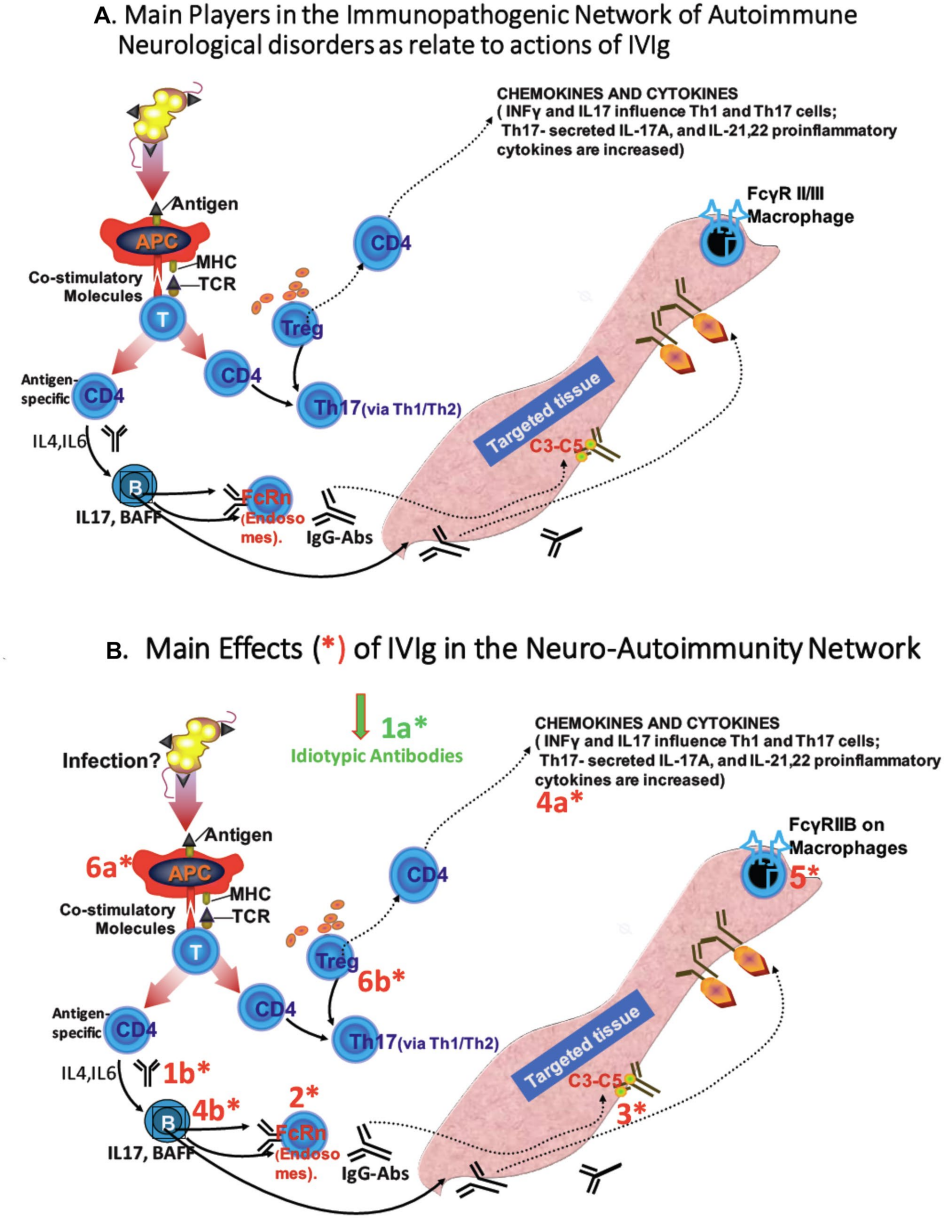
**A** Main players in the immunopathogenic network of autoimmune neurological disorders as relate to actions of IVIg. Nerve/muscle antigens, presented via APCs to CD4 + T cells via co-stimulatory molecules lead to upregulation of cytokines that stimulate B cells to produce IgG antibodies which, by fixing complement at the target organ, lead to tissue damage. Treg and Th17 + cells, cytokines such as IL-6 that affect the induction of Tregs, and proinflammatory cytokines, such as IL-17A, IL-21, and IL-22 are increased sustaining the immune imbalance. CD4 + T cells via IL-4, IL6 cytokines facilitate antibody production by B cells; the soluble B cell activation factor (BAFF) promotes B cell survival and maturation, while Treg and Th17 + cells affect antibody production via Th1/Th2 cytokine balance. FcRn plays a role in catabolism of IgG antibodies and Fcγ receptors on macrophages mediate inflammatory and immune effector functions (adapted from [171]). **B** Main effects of IVIg (1–6*) in the neuro-autoimmunity network. IVIg infuses idiotypic antibodies (1a*) and exerts multiple effects on: circulating autoantibodies (1b*); FcRn (2*); complement (3*); cytokines and proinflammatory molecules (4a*,b*), FcγRIIB receptors on macrophages (5*), and antigen-presenting cells, T-cell modulatory functions, and antigen recognition (6a*,b*)

In spite of the heterogeneity of the various autoimmune neurological diseases that respond to IVIg, generically almost every one of these disorders is variably mediated by antibodies, activated T cells, invaded macrophages, or complement (Fig. 2A, adapted from [171]). The mechanistic actions of IVIg, although predominantly target one or more of these processes according to the specific autoimmunity that governs each disease, also exert a collective effect on all relevant molecules associated with overlapping immune pathways. Collectively, the main effects of IVIg in the neuro-autoimmune networks include the following (Fig. 2B), as reported by many authors [1, 2, 5, 6, 9, 18, 23, 25].

#### Effect of Idiotypic Antibodies and Potential Neutralization of Pathogenic Autoantibodies (Fig. 2B, (1a*, 1b*))

The idiotypic antibodies within the IVIg (1a*) have the potential to bind to and neutralize pathogenic autoantibodies, thereby preventing their interaction with their autoantigens. This effect has been experimentally shown, when extracted F(αβ′)_2_ fragments of IVIg bound to and neutralized known autoantibodies, such as anti-DNA, anti-AChR, anti-thyroglobulin, anti-GM_1_, and others [11, 18, 26]. The idiotypic/anti-idiotypic effect has been confirmed in patients with GBS and some chronic demyelinating neuropathies whose serum contains various glycolipid antibodies against GM1, P0, GD_1a_, and GQ_1b_ and other glycoconjugates [27]. In an in vitro nerve-muscle preparation, idiotypic antibodies against these glycoconjugates naturally present within IVIg block and neutralize in minutes the “blocking” effect exerted by the serum of acute GBS patients on the quantal release [28, 29]. These idiotypic antibodies also exert a dose-dependent protection of cytotoxicity induced by GBS sera in an anti-ganglioside antibody-mediated cytotoxicity system [30]. Additionally, the F(αβ ‘)_2_, but not the Fc portion of the IgGs within the IVIg preparations, inhibits the binding of anti-GM1 antibodies to GM1-coated ELISA plates and the binding of cholera toxin to GM1 ganglioside [30], in a dose-dependent manner. If antiganglioside antibodies play a role in mediating myelin or axonal injury as proposed, their inhibition by IVIg can explain the noted clinical benefit [28–32]. The anti-idiotypic antibodies within IVIg may also affect antibody production by exerting negative signals on B cells when bound to antigenic determinants on B-cell surface (1b*) [33, 34]. IVIg also contains antibodies against CD_5_ molecules, which may suppress antibody production by affecting the autoantibody-producing CD20 + B cell subsets [35]. IVIg also reduces two important B-cell trophic factors, BAFF and APRIL, suppressing antibody production in experimental myasthenia [36]. Collectively, the multiple effects of IVIg on autoantibodies and B-cells are relevant in antibody-mediated autoimmune neurological diseases where IVIg is effective, such as myasthenia gravis, Lambert-Eaton myasthenic syndrome, antibody-mediated neuropathies, stiff-person syndrome, and NMOSD, as discussed below.

#### Acceleration of IgG Catabolism by Saturating the FcRn Transport Receptors: an Overlooked Factor Individually Affecting IVIg Dosing and Efficacy (Fig. 2B (2*))

**Fig. 3:**
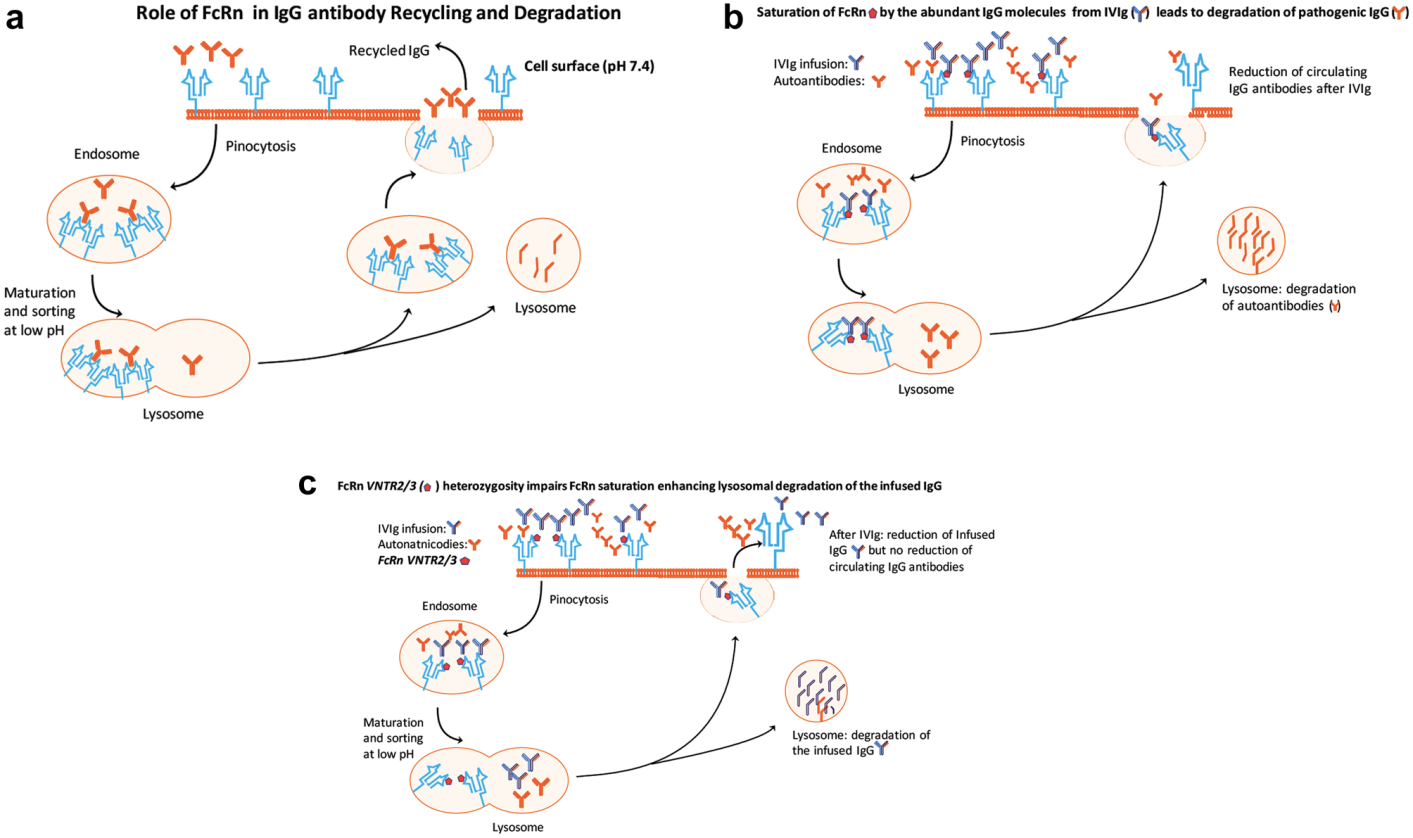
**A**–**C** Role of FcRn on recycling and degradation of pathogenic IgG antibody levels (**A**, **B**) and effect of FcRn heterozygosity (**C**) on sustainability of infused IVIg. **A** Normally, IgG antibodies (Y in red) bind to FcRn (light-blue receptors) to return, via the endocytotic vesicles, intact back into the circulation being protected by FcRn from degradation in the lysosomes. **B** The supra-physiological IgG levels from the infused IVIg (Y in blue) partially saturate the FcRn (saturated receptors depicted in red dots) leading to degradation of the endogenous IgG antibodies in the lysosomes, instead of being recycled back to the surface (degraded Y in red), resulting in reduced circulating IgG antibody levels. **C** The FcRn in heterozygous *VNTR2/3* patients (depicted with red dots on the light-blue FcRn receptors) may not be fully effective in saturating FcRn and protecting the infused IVIg from degradation; as result, part of the infused IgG is degraded in the lysosomes instead of fully returning back into the circulation (degraded Y in blue). In heterozygous *VNTR2/3* patients therefore, the IgG serum level from the infused IVIg does not increase as much as in homozygous *VNTR3/3* individuals who have fully functioning FcRn, resulting in lower immunomodulatory effect of IVIg and lesser reduction of the pathogenic antibodies; higher IVIg dose to compensate for its catabolism is perhaps needed for the *VNTR2/3* heterozygotes (adapted from [37])

IVIg can accelerate the catabolism of IgG antibodies by saturating the protective FcRn transport receptors found in many tissues but highly expressed on vascular endothelial cells. Normally, IgG antibodies bind to FcRn to return via endocytotic vesicles intact back into the circulation, being protected from degradation by the lysosomes (Fig. 3A) [37]. The supraphysiological levels of IgG derived from IVIg administrations saturate the FcRn so a portion of endogenously produced pathogenic IgG antibodies are not recycled back to the circulation but degraded [37, 38]; as a result, the infused IVIg, by competing with pathogenic autoantibodies for FcRn binding, reduces the serum half-life of autoantibodies by approximately 40% causing reduction of circulating IgG autoantibodies [37–40] (Fig. 3B). In randomized trials with IVIg in antibody-mediated diseases like stiff-person syndrome [41], Lambert-Eaton myasthenic syndrome [42] or EAMG [36], the respective anti-GAD, anti-VGCC or AChR antibodies titers are reduced, compared to placebo, within days after IVIg contributing to the noted clinical benefit, as discussed later.

The clinical importance of FcRn not only on efficacy of IVIg but also in individualized IVIg dosing has been strengthened by the impact of Variable Number of Tandem Repeat (VNTR) polymorphisms in the function of *FCGRT,* the gene that encodes FcRn [37]. Five VNTR alleles, (VNTR1-VNTR5), result in different expression levels of FcRn mRNA and protein level [37]; as a result, individuals homozygous for VNTR3 (VNTR3/3), the most common allele, have increased promoter activity and increased FcRn expression, compared to heterozygous VNTR3/2 individuals. Because high FcRn expression protects the infused IVIg from degradation, VNTR polymorphisms may affect IVIg efficacy by influencing the sustainability and increased serum IgG level of the infused IVIg. As discussed later, these polymorphisms do matter in certain individuals; VNTR2/3 heterozygous patients with Myasthenia Gravis treated with IVIg had shorter duration of infused IVIg due to fast IgG catabolism, lesser degree of AChR antibody reduction and poor response to IVIg, compared to VNTR3/3 homozygous patients [43] (Fig. 3C).

#### Inhibition of Complement Binding and Prevention of Membranolytic Attack Complex (MAC) Formation (Fig. 2B (3*))

The effect of IVIg on complement binding has been demonstrated in vitro, in animal models and in patients treated with IVIg. In early studies, IVIg was shown to prevent death in guinea pigs from the complement-dependent Forssmann shock by inhibiting the uptake of complement C_3_ and C_4_ fragments to the endothelial cells [44]. In patients with dermatomyositis, a complement-dependent microangiopathy mediated by activation of C3 and deposition of MAC on the endomysial capillaries [45], IVIg is not only clinically effective [46] but also inhibits complement uptake, intercepting the formation and deposition of MAC on the endomysial capillaries [46, 47]. In these patients, post-IVIg, but not post-placebo serum, inhibits the uptake of C3b and C4b fragments by sensitized in vitro targets, probably due to formation of covalent or non-covalent complexes between C3 and specific receptor sites within the infused IgG molecules [47]. Such an inhibition limits the available C3 molecules for further incorporation into the C5 convertase assembly, thereby preventing the formation and in situ deposition of MAC, as confirmed in the repeated muscle biopsy specimens of dermatomyositis patients treated with IVIg [46, 47]. Such an effect of IVIg on complement is a fundamental action of IVIg directly relevant to GBS, CIDP, and MG, where the complement pathway is activated and complement fragments are fixed in the targeted tissues [48, 49]. A complement-inhibiting and dose-dependent effect has been shown in an in vitro cytotoxicity assay in GBS patients when IVIg decreased the deposition of C3d complement fragments on the nerve fibers, preventing the cytotoxicity induced by the GBS sera [27–29]. In 39 CIDP patients, however, who participated in the largest controlled IVIg trial (ICE trial), the serum levels of the complement activation products C3a, C5a and soluble terminal complement complex (sTCC) were not modulated by IVIg but remained unchanged in patients who responded to IVIg, suggesting that the efficacy of IVIg in CIDP is based on immunomodulatory mechanisms different from complement inhibition [50].

#### Suppression of Pathogenic Cytokines and other Immunoregulatory Molecules (Fig. 2B (4a*, 4b*))

**Fig. 4:**
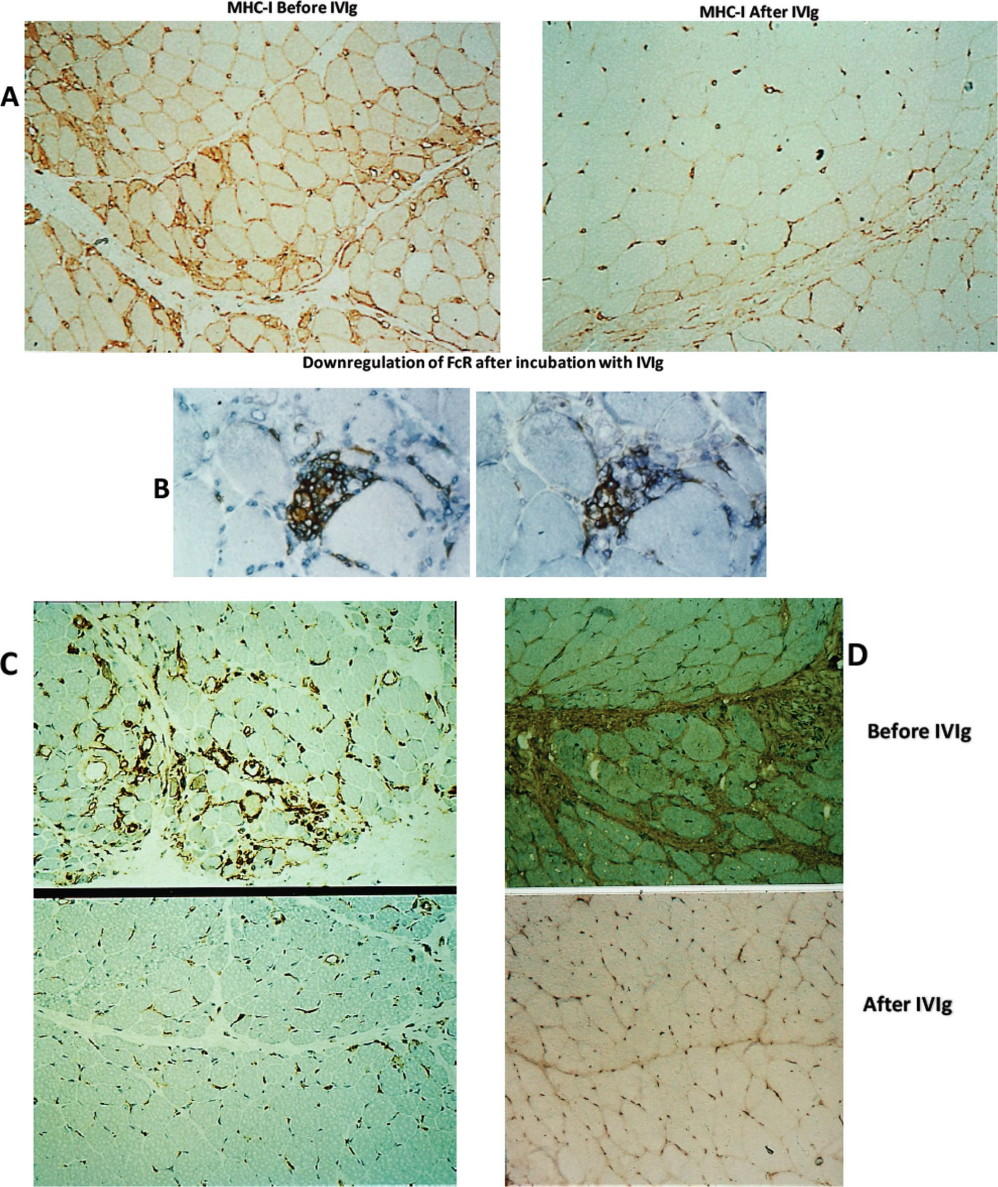
**A**–**D** Effect of IVIg on key Inflammatory mediators as observed in patients’ tissues after effective IVIg therapy (**A**,**C**,**D**) or on tissue sections (**B**). Downregulation of MHC-I (**A**), ICAM-1 (**C**), and TGF-b (**D**) in the repeated muscle biopsies from patients with dermatomyositis after effective IVIg therapy, compared to pre-treatment biopsies from the same patients; **B** downregulation of Fc receptors on endomysial macrophages in a patient’s muscle biopsy after incubation with IVIg [172]

Various proinflammatory molecules including cytokines, metalloproteinases (MMP-2, MMP-9), chemokines, chemokine receptors, and adhesion molecules such as IL_I_, TNF-α, IL_1β_, TGF-β, MHC-I, ICAM-I, LFA-1, MMP-2, MMP-9, CCR-2, CCR-4, CXCR-3, Mig, and IP-10 are increased in the circulation or are overexpressed on the endothelial cells, activated T-cells, nerves, or muscle tissues in patients with autoimmune neuropathies or myopathies [51–59]. IVIg causes a dose-dependent downregulation on their tissue expression or reduction in their circulating levels associated with clinical improvement [51–59]. In the repeated muscle biopsies of patients with dermatomyositis and on the lymphocytes or sera of patients with GBS who improved after IVIg [52, 53, 57, 59] there was impressive downregulation in a number of the aforementioned molecules including MHC-I, ICAM-I, LFA-1, TNF-α, IL_1β_, TGF-β, MHC-I, IP-10, Mig, MMP-2, and MMP-9 (Fig. 4A–D). Because upregulation of cytokines and chemokines is critical in the immunoregulatory pathways of all autoimmune neurological diseases responding to IVIg [1, 60], the downregulation of these molecules by the infused IVIg is pathogenetically relevant.

#### Modulation or Blockade of Fcγ Receptors(FcγR) on Macrophages (Fig. 2B (5*))

The FcγRs transduce either activating signaling via the FcγRIA, FcγRIIA, and FcγRIIIA or inhibitory signals via the FcγRIIB, regulating immune cell activation [2]. The IgG molecules bind through their Fc region to FcγR on hematopoietic cells, such as monocytes, macrophages, dendritic cells, and B cells, and mediate inflammatory or immune effector functions by activating or inhibiting intracellular signaling, cellular activation, proliferation, and cytokine production [61, 62]. IVIg suppresses the FcR expression on macrophages, as shown in tissue sections (Fig. 4B), and upregulates the low-affinity inhibitory FcγRII receptors on macrophages inhibiting phagocytosis and intercepting antibody-dependent cell-mediated cytotoxicity [18, 61–66, 68]. CIDP patients were found to have lower than normal FcγRIIB expression on naïve B cells and monocytes but after IVIg FcγRIIB expression was upregulated coinciding with clinical improvement [67]. It is likely that in GBS and CIDP, a blockade of the Fc receptors on macrophages by IVIg inhibits macrophage-mediated phagocytosis and intercepts the ongoing macrophage-mediated demyelination [60] resulting in clinical remission. Recent data indicate that genetic variations in *FCGR2*, the gene coding for FcγRIIb, affect the efficacy of IVIg in CIDP [68] as discussed later, supporting the importance of FcγRIIb in IVIg effectiveness.

#### Effects on Antigen-Presenting Cells, T-cell Modulatory Functions, and Antigen Recognition (Fig. 2B (6a*, 6b*))

IVIg inhibits the differentiation and maturation of dendritic cells and downregulates co-stimulatory molecules associated with cytokine secretion and antigen presentation [69–72], relevant to IVIg-responding autoimmune neurological diseases. IVIg also induces transient lymphopenia which is not dilutional, as demonstrated in controlled trials where lymphopenia was noted only after IVIg and not equal volumes of placebo [72]. IVIg also contains variable amounts of solubilized CD_4_, CD_8_, HLA-I, and HLA-II molecules that may interfere with antigen recognition by T-cells [26, 70]. Additionally, it contains antibodies that recognize and bind to highly conserved peptide sequences of the α_1_ helix of the HLA-I molecules [70] that may inhibit CD8-mediated cytotoxicity, a process relevant to several T-cell-mediated autoimmune neurological diseases. IVIg also inhibits T-cell apoptotic molecules like Fas and CD59 [73–75], enhancing further its inhibitory actions on activated T-cells.

#### Possible in Situ Effects in Muscle and Nerve Tissue

Because the blood-nerve barrier is defective at the roots and the distal nerve terminals, IVIg enters freely these areas and may exert an additional local effect directly on the respective tissues. As noted earlier, IVIg enters the endomysial tissue (Fig. 1B, C) and has been shown to exert a direct effect at the distal nerve terminals by blocking GM1 or GQ1b antibodies in vitro preparations [27, 28, 76, 77]. These effects are relevant to the efficacy of IVIg not only in inflammatory myopathies but also in acute motor axonal neuropathies and Miller Fisher syndrome where the GM1 or GQ1b antibodies bind at the nerve endings affecting neuromuscular transmission (76). Within the CSF the infused IVIg can enbathe nerve roots, but whether it can enter the endoneurial parenchyma and act in situ on endoneurial macrophages or complement-fixing antibody deposits has not been explored.

#### Effect on Immunoregulatory Genes

Genes for adhesion molecules, cytokines, MHC, chemokines, immunoglobulin, and structural proteins are variably upregulated in the muscles and nerves of patients with inflammatory myopathies and autoimmune neuropathies [78]. Among the most notable genes in inflammatory myopathies are the KAL-1 adhesion molecule, immunoglobulin genes, and genes for inflammatory mediators such as cytokines, chemokines, and interferon [78]. In CIDP, upregulated genes include tachykinin precursor which is relevant for pain; stearoyl-CoA-desaturase, a marker for remyelination; and the allograft inflammatory factor 1 (AIF-1), a modulator of immune response during macrophage activation [77]. Some of these genes have biological relevance, as demonstrated by microarray experiments on muscle biopsies obtained before and after IVIg therapy [78]. Muscles from patients with dermatomyositis who improved after IVIg [46] showed alteration of 2,206 genes; in contrast, 1700 of the same genes remained unchanged in sIBM who did not improve with IVIg (78). Among the markedly downregulated genes in DM, but not sIBM, were IL-22, KAL-1, ICAM-I, Complement C1q, and several structural proteins. The improved muscles from DM, but not sIBM patients, also showed upregulation of immunoglobulin-related genes and chemokines CXCL9 (Mig) and CXCL11, suggesting a role not only in immunoregulation but also in muscle remodeling and regeneration. Gene profiling in tissues and lymphocytes of patients with CIDP or GBS after IVIg may identify biologically relevant genes that could serve as biomarkers predicting response to therapy.

### Current Status of IVIg in Autoimmune Neurological Diseases: Efficacy Based on Controlled Clinical Trials

#### Guillain–Barre’ syndrome (GBS)

GBS is the most common acute demyelinating polyneuropathy causing weakness or paralysis of the limbs, bulbar, and respiratory muscles within 2–4 weeks from onset [79]. It follows viral or bacterial infections, including SARS-CoV-2 [80], being the most common post-viral autoimmune disease. Although the exact target antigen is unknown, molecular mimicry, antiglycolipid antibodies, T-cell sensitization, activated macrophages, and complement are the main immunopathological features of the disease [80]. In addition to the classic demyelinating form of GBS, there are GBS variants, including the acute axonal motor or sensorimotor forms, Miller-Fisher syndrome, or acute dysautonomic form [79]. Although IVIg appears helpful in all subsets, controlled studies have been conducted only in the classic sensorimotor demyelinating form.

Based on at least two randomized trials, one course of IVIg (5-day regimen of 0.4 g/kg/day) was comparable to plasmapheresis (PE) in outcome measures including time to unaided walking and discontinuation of ventilation [81]. Combining IVIg with PE or with 500 mg intravenous methylprednisolone showed no additional benefit [23, 79]. IVIg also remains the treatment of choice in childhood GBS leading to faster recovery and reduced morbidity, but controlled studies are not available and may never be conducted. GBS is a monophasic disease, but many patients remain with significant weakness within the first month of the disease, in spite of early IVIg initiation therapy. A small increase in serum IgG level 2 weeks after one IVIg infusion was independently associated with significantly slower recovery and more disability at 6 months [82], implying the need to test the benefit of higher dosage or an early second infusion. A second IVIg infusion in a small uncontrolled study in 38 selected patients with more severe disease, given within 1–2 weeks or 2–4 weeks from onset, did not show better outcome [83]. A large randomized, placebo-controlled trial was just completed in 327 GBS patients ≥ 12 years of age by the Dutch GBS study group [84]. On the first day, all 327 patients received the standard IVIg dose (2 g/kg over 5 days), but 93 of them with poor outcome (score of ≥ 6) at 7–9 days after inclusion were randomized to either a second 2 g/kg IVIg infusion over 5 days or placebo. At 4 weeks, the patients who received the second IVIg had more serious adverse events including thromboembolic complications, concluding that severely affected GBS patients with a poor early outcome not only do not benefit from a second IVIg course but may also have higher risk of adverse events [84].

#### Chronic Inflammatory Demyelinating Polyneuropathy (CIDP)

CIDP is characterized by slow onset (over weeks to months) weakness including proximal muscles, areflexia, and impaired sensation. Antibodies, activated T-cells, and complement have been implicated in the cause [85, 86]. A subset of patients, accounting for 10% of total CIDP, has IgG4 antibodies against nodal antigens, neurofascin-155 and contactin, forming an important subset when discussing IVIg effectiveness because most of them have IgG4 antibodies that respond poorly to IVIg [87].

Controlled studies have shown that steroids, plasmapheresis, and IVIg are equally effective on a short-term basis [85, 86, 88]. The ICE trial, a pivotal largest ever conducted in CIDP, has shown that IVIg is safe and effective not only for the short term but also for a long 48-week term leading to the first FDA-approved indication for a brand of IVIg [88]. A strong and positive effect on quality of life and improvement in some electrophysiological measurements was also noted [89, 90]. In most patients, IVIg becomes clearly effective after 6 weeks, necessitating the need for at least 2–3 infusions before concluding that it is ineffective [91, 92]. Although IVIg is generally considered as first-line therapy, the choice of how best to initiate therapy (choosing between prednisone, IVIg, or plasmapheresis which are both effective) is judged against cost, long-term side effects, patient age, venous access, disease severity, and concurrent illnesses [2]. Clinically, patients more likely to respond to IVIg are those with disease duration of less than a year, a relapsing course, no difference in strength between arms and legs, and electrophysiological signs of demyelination with conduction block [93]. FcγRIIB expression was found decreased in treatment-naïve CIDP patients and was upregulated after clinically effective IVIg therapy suggesting that FcγRIIB may be a factor predicting patients more likely to respond to IVIg [2]. Increased Fc glycosylation seems also associated with disease remission and response to IVIg, but the evidence is not sufficient to serve as disease biomarker [2].

##### Maintenance Therapy: is IVIg a Forever Therapy?

 CIDP, being the most common chronic neurological disease for which IVIg has been approved, has generated major challenges on how long to treat and at what doses [93–97]. Although maintenance dose was tested only up to 48 weeks in both, the ICE trial with 1 gm/kg IVIg every 3 weeks and the PATH study with weekly subcutaneous IgG [88, 97, 98], the optimal chronic maintenance regimen beyond that time-frame remains empirical, ranging among practitioners from 0.4 to 2 g/kg every 2–6 weeks for IVIg and 0.2–0.4 g/kg per week for subcutaneous IgG. The means of monitoring long-term efficacy or determining its necessity have also become quite variable, many times driven not by objective measures of dependency but by the patients’ assessment on how they subjectively perceive disease stability, weakness, pains, and fatigue. Evidence-based data and experience suggest that after the first 6–12 months of treatment, the need for continuing therapy should be based on dependency test utilizing the same objective measures used to determine initial efficacy, taking into account that patients’ anxiety in stopping IVIg may be a confounding factor [93, 94]. Apart from patients with continuing active disease who clearly improve after each infusion and worsen before the next, in our experience—shared also by others [94, 95]—many patients after 3–5 years have reached a long-term stability status of non-worsening (not necessarily remission) and may not need IVIg anymore; several patients have become convinced however that monthly IVIg is needed in ensuring stability and would have been worse without it. In a small placebo-controlled crossover trial in IVIg-dependent patients, more frequent lower dosing was not superior to low frequent high dosage in both efficacy and tolerance while serum IgG levels were irrelevant in determining the need for maintenance therapy [98]. Most enlightening about the necessity of IVIg in such patients is the data from 3 blinded controlled trials, where 40% of CIDP patients, being stable on monthly IVIg, did not relapse for 48 weeks when switched to placebo [88, 97, 99]. The major challenges of stopping therapy in chronic IVIg recipients, not only with CIDP but also with other diseases, are elaborated later.

Our preferred maintenance schedule has been based on the ICE trial and subsequent experience. We start with 2 g/kg per month for 3 months and re-examine the patient; if by then there is objective benefit, we start maintenance therapy combined with a dependency test. First, we lower the dose from 2 to 1 g/kg and re-examine the patient in 3 months. If the benefit is sustained, we prolong the intervals from 4 to every 5–6 weeks, in an indirect dependency test that diminishes the anxiety associated with complete stopping. According to the response based on neurological exam and AOL, we either resume 2 g/kg every 4–5 weeks or keep 1 g/kg every 3–4 weeks with subsequent dependency assessments every 6 months. Re-stabilization after stopping IVIg is always successful and patients are reassured that we will resume the previous schedule as soon as there are objective worsening signs.

#### Multifocal Motor Neuropathy (MMN)

It presents with a slow-onset weakness and muscular atrophy in the distal upper extremities, areflexia, preserved sensation, and conduction block of the motor axons. IgM antibodies to GM1 ganglioside are seen in up to 50% of these patients [100–103]. Unlike CIDP, MMN does not respond to steroids or plasmapheresis, but responds only to IVIg. Efficacy has been established with a number of controlled trials that led to FDA-approved indication [101]. The improvement lasts from 3 to 6 weeks, requiring maintenance re-infusions at almost predictable time periods. As symptoms diminish, the electrophysiological conduction block may resolve in some cases [103]. Therapy starts with 2 g/kg and maintained with 1 g/kg, following the pattern described for CIDP. In contrast to CIDP, however, where IVIg may not be needed in up to 40% of the patients after 5 years, in typical MMN, the IVIg is needed for much longer time periods with 80% of the patients requiring 1–2 g/kg monthly, although the optimal maintenance dose to arrest disease progression and prevent axonal degeneration remains also empirical.

#### Myasthenia Gravis (MG)

MG, characterized by fluctuating weakness or fatigability of the extraocular, bulbar, respiratory, and limb muscles, is mediated by pathogenic antibodies against the acetylcholine receptors (AChRs) in 85% of the patients; up to 5–7% of patients are seronegative, while another 5% have anti Musk antibodies [104−106]. Patients with MG respond fairly well to anticholinesterases, steroids, and immunosuppressants. Plasmapheresis is effective for crises or severe exacerbations; the same is true for IVIg in treating exacerbations in lieu of plasmapheresis. In two randomized trials, IVIg was as effective as plasmapheresis at day 15, either at 1 g/kg or 2 g/kg [107–110]. IVIg is also superior to placebo, 14 days after therapy, in patients with moderate to severe MG and “worsening weakness” [110]. A recent prospective non-randomized study in 49 patients with MG exacerbations confirmed the efficacy of 2 g/kg of IVIg observed 14 days after the infusion, but without any new data beyond the 30-day period [110]. The AChR antibody titers may decline or remain unchanged; in vitro, IVIg inhibits AChR antibody activity up to 30% of the preincubation level [31]. Subcutaneous IgG is also effective in uncontrolled series for relapses up to 4–6 weeks [111]. At present, IVIg is justified in lieu of plasmapheresis for acutely worsening disease to prevent or minimize impending bulbar or respiratory failure, prepare a clinically weak patient for thymectomy or on a short-term basis to help significant worsening. The role of IVIg in the chronic management of MG or as a steroid-sparing agent has not been established in prospective randomized trials [112, 113]. Unfortunately, IVIg is often being off-label used, in lieu of steroids and immunosuppressants, for chronic therapy to maintain stability or reduce the uncertainty of possible exacerbations without evidence of cost-effective benefit. Considering its ineffectiveness in IgG4 antibodies, IVIg is not substantially helping Musk-positive MG because it has no effect on IgG4 antibodies as discussed later. IVIg has been effective in Lambert-Eaton myasthenic syndrome, based on a small placebo-controlled study that improved muscle strength compared to placebo 2–4 weeks after therapy [42]. No long-term benefits have been established in controlled studies.

#### Inflammatory Myopathies

The main subsets in this large family of diseases include dermatomyositis (DM), necrotizing autoimmune myositis (NAM), overlap myositis, and inclusion body myositis (IBM) [45, 114]. Polymyositis is so rare that its existence as a distinct entity is now questioned. Dermatomyositis presents with proximal muscle weakness and a violaceous rash on face and extremities caused by early deposition of membranolytic attack complex (MAC) on the endomysial capillaries that leads to capillary destruction, muscle ischemia, and inflammation [114]. NAM causes severe and often acute muscle weakness due to extensive macrophage-mediated muscle fiber necrosis. IBM is a chronic disease with slowly progressive proximal and distal weakness along with muscle atrophy caused by a combination of T-cell-mediated cytotoxicity along with a degenerative process associated with protein misfolding [114].

In a pivotal placebo-controlled study, IVIg was effective in dermatomyositis resulting in significant improvement of muscle strength and skin rash and resolution of muscle histology and immunopathology. Clinical benefit corresponded to significant improvement in muscle cytoarchitecture, increased muscle fiber diameter, reduction of inflammation, revascularization, interception of complement deposition, and downregulation of inflammatory mediators at the protein, mRNA, and gene level [46, 47, 53, 57]. A new large-scale randomized, placebo controlled, phase III study (ProDERM: Progress in DERMatomyositis) evaluating the efficacy and long-term tolerability of IVIg (Octagam 10%) up to 16 weeks with a 24-week extension phase has been now completed [115] and led, as of July 15, to FDA approval for this brand of IVIg for dermatomyositis [FDA orphan drug approval—Octapharma’s Octagam 10% receives 7 years of market exclusivity for adult dermatomyositis. Octapharma AG. 2021 Aug. 30]. IVIg is also effective in some patients with NAM [114], but a controlled study has not been performed. IVIg is ineffective in IBM based on two controlled studies; it did however statistically improve the patients’ dysphagia [116] and may be considered for life-threatening dysphagia for short time periods.

##### Retroviral-Inflammatory Myopathies

PM and IBM can be seen in association with human immunodeficiency virus and human T-cell lymphotropic virus-I infection with the virus triggering a T-cell-mediated inflammatory response identical to retroviral-negative PM and IBM (114). In 3 PM patients we treated in a controlled crossover design similar to the one used for DM (46), only minimal changes in the patients’ strength were noted compared with placebo, terminating the study early [31].

#### Stiff Person Syndrome (SPS**)**

SPS is a disabling autoimmune disorder characterized by muscle stiffness, episodic muscle spasms, phobias, and antibodies to glutamic acid decarboxylase (GAD65). In a placebo-controlled, crossover study, the efficacy of IVIg was assessed with validated stiffness index and heightened-sensitivity scales [41]. Among patients initially treated with IVIg, stiffness scores decreased significantly (*p* = 0.02) and heightened-sensitivity scores declined markedly, but all rebounded during placebo; the opposite occurred among those treated with placebo first [41]. Patients who received IVIg compared to placebo were able to walk without assistance or falls and perform daily activity functions. The study showed that IVIg is effective in SPS patients, not adequately responding to anti-spasmodic and GABA-enhancing drugs, for up to 3 months [41]; its long-term monthly maintenance therapy for chronic SPS management has not been however tested in a controlled study.

#### Autoimmune Encephalitis (AE), Autoimmune Epilepsy, and LGI1/CASPR2 Autoimmunity

AE presents with acute or subacute onset of aberrant behavior, psychosis, agitation, depression, sleep disturbances, seizure-like phenomena, intractable seizures (autoimmune epilepsy), movement disorders, memory changes, or coma [117, 118]. The syndromes are highlighted by autoantibodies against synaptic antigens and receptors (i.e., NMDAR, GABAa, GABAb, AMPA, and glycine receptors), proteins that stabilize voltage-gated potassium channel complex into the membrane (like the leucine-rich, glioma-inactivated-1 (LGI1) and contactin-associated protein-like 2 (CASPR2)) and enzymes that catalyze the formation of neurotransmitters such as GAD [17]. AEs are potentially treatable responding to immunotherapies with steroids and IVIg followed by plasma exchange and anti-B cell agents. The efficacy of IVIg remains strong based on clinical observations but not randomized trials due to obvious difficulty enrolling acute encephalopathic patients. In spite of many challenges however, a double-blind placebo-controlled IVIg trial was conducted in LGI1/CASPR2-associated autoimmune epilepsy in 17 patients (14 with LGI1; 3 with CASPR2) having ≥ 2 seizures per week [119]. Over a 34-month study period, the IVIg-randomized group had a statistically significant reduction of seizure frequency compared to placebo with some patients becoming seizure-free at the end of the blinded phase. The study concluded that IVIg was effective with good safety profile compared to the frequent side effects observed with antiepileptics [119]. Although the anti-LGI1 and anti-CASPR2 IgG antibodies are predominantly of IgG4 subclass, the IgG4 predominance did not seem to affect the IVIg efficacy although IgG subtypes were not systematically analyzed. IVIg has been also effective in COVID-19-inducced, presumably autoimmune, status epilepticus [120].

#### Neuromyelitis Spectrum Disorder (NMOSD) with Anti-AQP-4 or Anti-MOG Antibodies

Aquaporin-4 (AQP4) IgG-positive and myelin oligodendrocyte glycoprotein (MOG)-IgG–associated disorder (MOGAD) are CNS demyelinating diseases that present with optic neuritis, and transverse myelitis; some MOGAD patients may also have in combination acute disseminating encephalomyelitis or brainstem encephalitis. The diseases are either monophasic or relapsing with highly variable frequency of recurrent demyelinating attacks requiring long-term immunosuppressant therapy with mycophenolate, rituximab, azathioprine, or IVIg. In a large retrospective multicenter study in MOGAD patients, maintenance therapy with IVIg reduced recurrent demyelinating episodes and induced the lowest median annualized relapse rate (ARR) [121]. Maintenance IVIg (at 3- or 4-week intervals) was associated with the greatest reduction in relapse rate with only 20% of patients having a relapse, in contrast to > 50% receiving other therapies, concluding that IVIg was more effective in suppressing attacks [122]. The efficacy of IVIg in NMOSD as rescue treatment and reducing relapse rate was also compared between intravenous methylprednisolone (IVMT) or IVMT + IVIg. Patients treated with IVMT + IVIg for 17.39 ± 2.75 months exhibited longer time to next relapse compared to patients on IVMT; the superiority of IVIg as rescue treatment and inducing longer remissions was especially prominent in AQP4-ab-seropositive patients. A retrospective review of NMO-IgG-positive patients treated with IVIg (0.4 g/kg/day) every 1–3 months in combination with azathioprine showed that in 95% of patients, the add-on IVIg significantly reduced the median ARR with 35% of patients being relapse-free during a mean period of 43.5 months, and 80% without disability progression; the median NMO-IgG titer decreased after treatment and became negative in three patients [123]. In an online survey of clinicians treating MOGAD, treatment approach was highly variable, but for children, IVIg was the preferred first-line therapy by 54.5% [124].

#### Neuropathic Pain Syndromes: Possibly-autoimmune Small Fibre Sensory Neuropathy (SFSN), Reflex Sympathetic Dystrophy (RSD), and Complex Regional Pain Syndrome (CRPS)

This is a heterogeneous neuropathic pain syndrome with overlapping symptomatology. The rationale for considering IVIg is based on the observation that in some of them, the pain syndrome co-exists with systemic autoimmunities, while in others proinflammatory cytokines or antibodies to non-specific antigens have been implicated [125]. The efficacy of IVIg is variable, or even considered controversial, with some negative and other promising small trials.

*SFSN* is now one of the commonest neuropathies. Patients present with diffuse pains; intolerance to light touch, allodynia, or hyperalgesia; and some with autonomic features while others overlap with fibromyalgia and erythromelalgia [126, 127]. They have normal neurological examination including sensation, reflexes, balance, and electrophysiological studies but reduced intraepidermal nerve fiber density on skin biopsy. Most importantly, 20% of patients may have a systemic autoimmune or rheumatic disease, such as Sjogren’s syndrome, celiac disease, rheumatoid arthritis, or non-specific immunological abnormalities [126–128] and can respond to IVIg based on clinical observations [128]. Two non-specific autoantibodies, one against *trisulfated heparin disaccharide (TS-HDS)* and another against *fibroblast growth factor-3 (FGFR3*), have been detected more frequently than in controls [129]. A small placebo-controlled trial of IVIg in 60 SFSN patients without underlying autoimmune conditions [130] showed that after 3 months, the pain improved in 40% of IVIg-recipients compared to 30% of the placebo, concluding no clinically relevant benefit. The study was however centered on the economics of IVIg, not taking into consideration the economics of disability induced by SFSN or patient subsets with immune etiologies that can benefit from IVIg for some time period.

In *RSD*, a small placebo-controlled-crossover trial examined the efficacy of low-dose IVIg 0.5 g/kg in 12 patients 6–19 days after IVIg [131]. Although a reduction in pain scales was noted, the study was too small to be conclusive. In *CRPS*, pain is due to an assumed defect in the sympathetic–sensory decoupling mechanism supported by IgG autoantibodies against β2-adrenergic receptor (β2AR) or muscarinic-2 receptor (M2R) observed in some patients. Because both β2AR and M2R are involved in modulating pain and inflammation [132] and M2R autoantibodies are capable of promoting pain through nociceptive hyperexcitability, IVIg may be a reasonable treatment option, as shown in some anecdotal reports. Collectively, considering the increased frequency of all these painful syndromes and the significant disability they induce, vigorously controlled large-scale trials are needed to conclusively establish if IVIg exerts any benefit, in which subset and for how long.

#### Post-polio Syndrome

This is a chronic degenerative condition clinically characterized by new muscle weakness, fatigue, and pain that develop many years after the initial attack of acute paralytic poliomyelitis [133]. It is thought to be due to attrition of the surviving motor neurons [133, 134]. Lymphocytic infiltrates in the patients’ spinal cords have been however observed even 30 years after the original infection, and upregulation of mRNA for tumor necrosis factor (TNF), IFN-γ, and interleukins (IL-4 and IL-10) has been observed in the CSF, raising the possibility of a persistent smoldering inflammatory response [134, 135]. Following IVIg treatment, the CSF IFN-γ and TNF mRNA levels were reduced prompting a controlled trial in 135 post-polio patients. Although the results were of uncertain clinical importance, the trial showed statistical significant changes in some physical activity and quality of life scores [135], prompting a still ongoing FDA-approved phase II-III clinical trial.

#### IgM MGUS-anti-MAG-neuropathy

Prompted by improvement observed in 2 such patients after IVIg [136], a controlled study was conducted in 11 patients. Modest but not statistically significant benefits were observed; strength improved in 2 of 11 patients and declined after placebo, while sensory scores improved in a third [137]. Antibody titers to MAG/SGPG did not appreciably change. Similar results were obtained in a European trial. An inverse relationship between degree of IgM binding to MAG/SGPG and IVIg responsiveness was noted with those few responders having the lowest strength of reactivity [138].

#### Alzheimer’s Disease (AD)

Abnormal accumulation of β-amyloid is a ubiquitous and early event in the pathogenesis of AD. Because IVIg contains natural anti-β-amyloid antibodies and in AD murine models IVIg resulted in minimal plaque clearance, its efficacy was explored in AD patients [139]. After several small studies showing promising benefits, a large phase-III placebo-controlled trial was conducted in 390 patients with mild to moderate AD randomized to either placebo (low-dose albumin) or two doses of IVIg 0.2 or 0.4 g/kg every 2 weeks for 18 months [140]. The primary outcome was change in cognitive AD-Assessment Scale and functional AD-Activities of Daily Living Inventory from baseline to 18 months. No beneficial effects were observed [140]. Interestingly, significant decreases in plasma Ab42 (but not Ab40) levels were observed in IVIg-treated participants.

#### Miyoshi’s Myopathy

Based on the rationale that Miyoshi’s myopathy is a necrotizing myopathy with muscle fiber necrosis mediated by MAC, a double-blind, placebo-control, crossover trial of monthly IVIg was performed in 3 patients for 6 months, following the protocol we used in IBM patients [116]. No objective changes in muscle strength were noted after 7 months of treatment (Dalakas MC, Sivakumar K, Spector S, unpublished observations, 1993–1996) terminating the study early [31].

## Small Uncontrolled but Clinically Important IVIg Trials

- **Amyotrophic Lateral Sclerosis (ALS).** In a study of 9 patients, IVIg treatment failed to change the course of the disease [141].
- **Duchenne Muscular Dystrophy (DMD).** Because DMD patients respond to steroids, have endomysial inflammation, and the muscle fiber necrosis is mediated by MAC activation, a prospective open-label pilot study was performed in 10 patients monthly for 6 months, monitoring disease progression with quantitative muscle strength testing, MRC scales, functional scores of timed activities and stair climbing, and video-recording of walking. After 6 months, 5 patients continued to worsen, but the other 5 stabilized or seemed slightly improved, based on objective changes in the activities of daily living and quantitative muscle strength scores. The 5 seemingly stable or slightly improved patients continued to receive IVIg treatments for 1 year and 3 of them for up to 2 years. Although in 3 patients there seemed to be stability or even improvement, when the patients’ course was compared with the DMD natural history data, there was not enough evidence to conclude that IVIg had definitely changed the disease course to justify a controlled study [31] (Dalakas MC, Sekul EA, Koffman BM, unpublished observation, 1992–1996).
- **Narcolepsy.** In narcolepsy, there is hypocretin (also known as orexin) deficiency thought to be of autoimmune mechanism because of linkage to human leukocyte antigen genes. In four patients with hypocretin-deficient narcolepsy, IVIg improved within a few months the frequency and severity of cataplexy, based on repeated polysomnographies and specific questionnaires [142, 143]. Beneficial effects, with greater than 90% reduction in frequency of cataplectic attacks, persisted in three patients for at least 2 years without any additional IVIg treatment. Other trials however showed only mild but not persistent benefits in some patients [142, 143].

## IVIg in IgG4 Neurological Diseases

Several studies have repeatedly shown limited efficacy of IVIg in neurological disorders mediated by IgG4 pathogenic antibodies, a non-complement-fixing IgG antibody subclass. The main IgG4 antibodies in autoimmune neurology are (a) *MuSK IgG* in myasthenia, (b) nodal/paranodal *Neurofascin-155* and *CASPR1 IgG* in CIDP, (c) *LGI1-* and *CASPR2-IgG* in autoimmune encephalitis with IgG 1:4 ratio reported to correlate with clinical outcomes, and (d) *anti-IgLON5* associated with a possibly autoimmune neurodegenerative process, presenting with bulbar syndromes, sleep apnea, and REM-sleep behavior disorder. The reasons why IVIg is not effective in IgG4-syndromes relate to IgG4 being non-inflammatory and non-complement-fixing antibodies making all the described anti-inflammatory and complement-inhibitory effects of IVIg irrelevant; further, IVIg contains IgG with less than 5% of IgG4 subclass that cannot exert any neutralization effects on the pathogenic IgG4 antibodies [144].

## **Genetic Factors Affecting IVIg Efficacy and Dosing:***FcRn Polymorphisms andFcγRIIb***Genotypes**

### VNTR Polymorphism and Outcome of IVIg Therapy

As mentioned earlier, the supraphysiological levels of IgG derived from IVIg administrations saturate the FcRn ensuring high serum levels of infused IgG [37] (Fig. 3B). The FcRn is however functionally more effective in homozygous VNTR3/3 patients offering higher protection of the infused IgG compared to heterozygous patients with VNTR3/2 polymorphisms where part of the infused IVIg is degraded [37] (Fig. 3C). Pharmacokinetic data from 174 patients with Guillain–Barre syndrome have shown that 2 weeks after one IVIg administration, the patients with higher increase in circulating IgG had significantly better outcome [82]. In a limited retrospective analysis however, no relation was found between VNTR3/3 polymorphism and outcome [145]; similar non-significant correlations were also noted in 23 patients with multifocal motor neuropathy [37]. In a recent large study in 334 patients with MG treated with IVIg, the patients with VNTR2/3 genotype had significantly shorter retention of the infused IVIg due to faster IgG catabolism, lesser degree of AChR antibody reduction, and poor response to IVIg compared to VNTR3/3 homozygous patients [43], suggesting that polymorphisms do matter in IVIg efficacy in certain patients by affecting the level and sustainability of the infused IgG (Fig. 3C). This unexplored concept may potentially answer the fundamental question as to why in all effective randomized controlled studies in patients with CIDP, GBS, MMN, MG, dermatomyositis, or stiff person syndrome mentioned earlier, 20–30% of the patients do not respond to IVIg even if their disease status is identical to that of responders [37]. If IVIg ineffectiveness in some patients is due to VNTR genotypes, higher IVIg doses or more frequent administrations may be needed. We all have seen patients infused with 3 g/kg per month to achieve effectiveness, with a recent series of 6 CIDP patients responding only to super-high IVIg doses, up to 4–6 g/kg per month [146].

### Genetic Variations in the Gene Coding for Fc**γ**RIIb (FCGR2B)

These genetic variations, linked to development of autoimmune disorders, such as Kawasaki or CIDP, are also connected to IVIg response [51, 61–63, 65–68]. In a recent study, patients with certain *FCGR2B* promoter variants responded more often to IVIg than patients with other variants [68]. The Fc**γ**RIIb receptor, expressed by monocytes, macrophages, dendritic cells, and B cells, is a negative regulator of cellular activation, proliferation, and cytokine production, but it is upregulated by IVIg [61–63, 65–68]. The concept that Fc**γ**RIIb genotypes affect Fc**γ**RIIb expression and may play a role in the efficacy of IVIg should be explored in future treatment trials.

## SCIg

Subcutaneous IgG (SCIg) can be an attractive route of administering IgG for treating patients with autoimmune neurological diseases because it can ensure a steady-state IgG level avoiding end-of-dose effects, while providing the convenience of self-administration [147]. The efficacy of SCIg has been documented in CIDP, MMN, and dermatomyositis [148–150]. The PATH study, the largest controlled 24-week CIDP trial, showed that SCIg was efficacious as maintenance therapy in IVIg-dependent patients [97]; it did not however establish whether SCIg is as good or preferable to IVIg because no head-to-head comparison was done [150]. Importantly, 26% of SCIg-receiving patients relapsed, indicating that switching a stable IVIg-receiving patient to SCIg may carry a relapse risk. Further, 21% of patients withdrew for reasons unrelated to relapses, raising the possibility of frustration or discomfort with pumps, tubes, or injection-site reactions [150]. For patients with poor venous access, cardiovascular risks, or systemic IVIg-related side effects, switching to SCIg is recommended. Overall however, the choice comes to personal preference that needs to be tested: 1–2 days per month IVIg infusions at home or outpatient clinics, or four weekly home self-infusions (finger strength permitting) in two to eight parallel sites of 50 mL each, lasting 1–2 h that ensures independence and home-convenience? Time will tell. SCIg is preferred in some counties for financial or practical reasons because home infusion companies are not allowed; direct comparative data on efficacy, tolerance, patient preference, and economics are not presently sufficient to recommend a priori one of the two treatment approaches. For patients stable on IVIg, switching to SCIg should be a shared decision with the patients discussing on a case-by-case basis IVIg intolerance, end-of-dose effect, venous access, and convenience. Some of our patients were unhappy switching from IVIg to SCIG and preferred a port insertion rather than SCIg, but others were much happier switching to SCIg.

## Stopping Chronic IVIg Maintenance Therapy: a Challenging Issue When IVIg is Perceived as a “Forever” Treatment

In the 3 largest placebo-controlled CIDP trials, ICE [88], PATH [97], and FORCIDP [99], 40% of patients receiving chronic IVIg for disease stability did not relapse over a 24-week period when randomized to placebo, concluding that placebo is an important variable in patients on chronic maintenance therapy, especially older patients with residual deficit. We are observing similar responses in some SPS and MG where IVIg is extensively used “off-label” for chronic maintenance therapy. It has now become clear that in several of these patients, the chronic “stability status” expressed as “non-worsening” attributed to monthly IVIg is not IVIg-related but rather a *conditioning effect*, *learned response* and *expectation*, that collectively account for a *placebo/nocebo* phenomenon, often observed with chronic intravenous therapies [94, 151]. We witnessed similar effects in the placebo-controlled trial with rituximab in SPS, where quality of life assessments after 6 months were statistically significant in both, the rituximab and placebo-randomized patients, leading to a negative trial [152]. One of the main challenges in these patients is how to interpret the IVIg-dependency test we all perform by either prolonging infusion intervals, reducing monthly dose or stopping therapy; apart from around 60% of patients who demonstrate objective signs of worsening while off IVIg, about 40% of patients remain unchanged on objective neurological assessments but express subjective symptoms of increased fatigue, pains, spasms, and various heightened bodily attentions collectively communicated as being less able to perform daily tasks due to “disease worsening.” Such patients strongly wish to return to their previous schedule, becoming often upset when IVIg is altered; they are justifiably fearful however, when recall events of their original illness several years back, like GBS paralytic events, MG crises, and severe spastic events in SPS and believe that monthly IVIg protects them from such recurrences. This is not a placebo effect but a combination of conditioning, fear, effects of various psychological, environmental, emotional, or aging factors and uncertainties of present life events that collectively built the conviction that monthly IVIg ensures stability. It is like the biopsychosocial model discussed for the functional neurologic disorders [153]. How do we approach such a *conditioning/learned* effect? Although there is no answer, it is prudent to advice new patients from the outset that: (a) will try IVIg for 3 months; if no meaningful or objective benefit will stop; (b) if there is benefit, we will continue at half the dose to objectively assess maintenance dose and infusion intervals; (c) IVIg is not a forever therapy and periodic dependency tests are needed to objectively document disease worsening if IVIg is reduced or stopped; d) reassure the patients that re-stabilization is always effective after resuming the previous schedule, if objective signs of worsening; and e) there is no scientific evidence justifying a forever IVIg therapy especially when emerging age-related co-morbidities raise safety concerns. A survey of 100 US community neurologists treating CIDP showed that one out of 3 do not discuss weaning off IVIg; only 39% inform their patients that will discontinue it at some point, while 1 of 10 said will give IVIg for life; they believe however that 38% of their patients can be weaned off IVIg [154].

## IVIg-Related Practical Issues: Dosing, Infusion Reactions, Complications, and Laboratory Abnormalities

### Current Dosing

The therapeutic dose of IVIg is empirically set at 2 g/kg followed by a maintenance monthly dose of 1 g/kg, as dictated by efficacy and duration of clinical benefit [155, 156]. This can be administered into two daily doses although in older people and those with impaired renal or cardiovascular function, the total dose can be split over 3 to 4 days. A dose-finding efficacy study for a given disorder has not been however performed until now when a randomized, phase III study in patients with CIDP (ProCID trial) compared the efficacy and safety of 3 different IVIg doses (0.5, 1.0, and 2.0 g/kg, 10% IVIg (panzyga)) every 3 weeks for 24 weeks in 142 enrolled patients [157, 158]. The primary efficacy endpoint was the response rate in the 1·0 g/kg group. After 24 weeks, the response rate was higher in the 1 g/kg group demonstrating efficacy, but the response rate in the 0.5 and 2 g/kg groups did not differ significantly from the 1 g/kg group [158]. These interesting results raise fundamental questions about how maintenance is defined, the minimum clinically effective dose considering that 0.5 g/kg exerts reduced immunomodulatory effects in basic experiments, and the significant concerns with placebo/nocebo effects while on chronic therapy as noted above.

A concern of calculating the efficacy dose based on ideal vs total body weight has not been studied either but based on expert opinions is set on ideal body weight, although the opinions of practicing neurologists are variable [154, 159].

### Infusion-Related Reactions

These are usually minor, occurring in not more than 10% of the patients and include mild to moderate headache that responds to non-steroidal anti-inflammatory medications, fever, chills, myalgia, chest or back pain, and temporary rise in blood pressure during the first hours of the infusion; they usually respond by stopping the infusion for 30 min and resuming it at a slower rate [1, 2, 9, 23, 24]. Post-infusion fatigue, fever, or nausea may also occur lasting for 24–48 h. Skin reactions can develop 2–5 days after the infusions, may last up to 30 days, and include urticaria, lichenoid cutaneous lesions, pruritus of the palms, and petechiae of the extremities. Skin reactions, associated with various IVIg lots, occurred in seven of 120 patients we had initially treated [1]. In a large retrospective evidence-based study of all trials performed in neurological disorders, headaches occurred in 16.6%, fever 6.6%, hypertension 4.6%, chills 3.3%, and nausea in 3.2% [159]. Some patients develop severe headache due to aseptic meningitis as first reported in several of our patients, being unrelated to the proprietary product, rate of infusion, or underlying disease [24, 160, 161]; prophylaxis with intravenous steroids can be occasionally helpful, but symptoms respond to strong analgesia and subside in 24–48 h. Additional diagnostic testing is rarely necessary. IVIg therapy may also trigger a migraine attack in patients with prior history; aseptic meningitis is also higher in migrainous patients [24, 161].

There is a remote potential for a severe anaphylactic reaction due to absence or severe deficiency of IgA in patients who also have anti-IgA antibodies. This rarity occurs in patients with common variable immunodeficiency [162]. IgA deficiency is common in the general population (prevalence about 1:1000), but it is not a risk factor by itself; about 29% of these individuals have anti-IgA antibodies, but their presence does not necessarily predict the development of allergic reaction from IVIg [163].

### Adverse Reactions During Chronic Use

Rare cases of thromboembolic events such as strokes, pulmonary embolism, or myocardial infarction have occurred after IVIg treatment [164−167]. One causative factor may be the increase in serum viscosity, especially in patients with risk factors, such as cryoglobulinemia, hypercholesterolemia, or hypergammaglobulinemia [165, 166]. Patients with recent deep vein thrombosis, or immobilized patients who may have a subclinical thrombosis, may be at higher risk. If IVIg is highly efficacious and is needed to ensure adequate physical functioning, it can be given to patients on anticoagulants, under close supervision and special adjustment of anticoagulant therapy. Acute renal tubular necrosis, mostly reversible, occurs rarely with IVIg therapy in patients who have pre-existing kidney disease and volume depletion, especially the elderly and those with diabetes or poor hydration. It is usually reversible, but very rare serious events have been noted [168, 169]. Serum creatinine may rise 1–10 days of the infusion but returns to baseline within 2–60 days of discontinuation. This complication has been more often associated with the high concentration of sucrose in some proprietary IVIg products. Osmotically induced tubular injury and vacuolization have been noted on renal biopsy [169]. In patients with pre-existing kidney disease, close monitoring of creatinine and BUN is therefore essential while slowing the rate of infusion or selecting low osmolality products minimizes the risk. Among the various IVIg preparations on the market, there is no documented evidence that one is more efficacious than the other for a given disorder. Some products may however be preferable for certain high-risk patients if low osmolality, low-sodium, or low-sucrose deem essential. Overall, IVIg is considered safe for long-term administration, as used in autoimmune neuromuscular diseases, but need to be mindful for emerging comorbidities.

### Laboratory Abnormalities

After IVIg therapy, the erythrocyte sedimentation rate increases sixfold [31, 73] due to enhanced rouleaux formation and reduced surface area caused by the infused gamma globulin. This false increase can persist for 2 to 3 weeks and should not be considered a sign of a developing autoimmune inflammatory process. Hyponatremia, as low as 130 mg/l (normal, 135–145 mg/l), after IVIg therapy can be also detected due to the assay method used to measure Na^+^ that requires additional dilution of the sample owing to the high serum protein concentration after IVIg infusion [31, 72]. A mild and inconsequential leucopenia can be also observed [72]. IVIg contains natural autoantibodies, as mentioned earlier, which can be detected up to 30 days after the infusion requiring increased awareness not to misdiagnose autoimmunity especially if the initial treatment was empirical.
